# Supplementary material for: Semantic Self-Images and Well-Being in Young and Older Adults: Does the Accessibility Matter?
Source: Brain Sci. 2022 May 31;12(6):716. doi: 10.3390/brainsci12060716 (PMC9221324; doi:10.3390/brainsci12060716)
Supplement: Supplementary file 1 [file brainsci-12-00716-s001.zip › brainsci-1732651-supplementary.pdf]

SUPPLEMENTARY MATERIALS

Emotional Valence

Table S1. Results of the multinomial regression for the emotional valence of the first statement

Predictor	Estimate	SE	z-statistic	P	r
		Negative as reference			
Neutral vs Negative					
(Intercept)	1.27	1.34	0.95	.341	.11 [-.12, .33]
Group(Young)	0.77	1.93	0.40	.688	.05 [-.18, .27]
PSS	-3.30	2.06	-1.60	.109	-.18 [-.40, .04]
PANAS-NEG	3.00	2.22	1.35	.177	.15 [-.07, .37]
PANAS-POS	-1.68	1.70	-0.99	.324	-.11 [-.34, .11]
SWLS	2.14	1.70	1.26	.206	.14 [-.08, .37]
Group(Young):PSS	1.93	2.99	0.65	.519	.07 [-.15, .30]
Group(Young):PANAS-NEG	-2.17	2.55	-0.85	.395	-.10 [-.32, .13]
Group(Young):PANAS-POS	2.54	2.10	1.21	.225	.14 [-.08, .36]
Group(Young):SWLS	-1.00	2.15	-0.47	.642	-.05 [-.28, .17]
Positive vs Negative					
(Intercept)	2.66	1.19	2.24	.025	.25 [.04, .46]
Group(Young)	-0.47	1.84	-0.26	.797	-.03 [-.26, .20]
PSS	-3.01	2.01	-1.50	.134	-.17 [-.39, .05]
PANAS-NEG	4.42	2.28	1.94	.053	.22 [.00, .43]
PANAS-POS	-0.82	1.69	-0.48	.629	-.06 [-.28, .17]
**SWLS**	**3.68**	**1.86**	**1.98**	**.048**	**.22 [.01, .44]**
Group(Young):PSS	1.06	2.98	0.36	.721	.04 [-.19, .27]
Group(Young):PANAS-NEG	-4.49	2.65	-1.70	.090	-.19 [-.41, .03]
Group(Young):PANAS-POS	2.49	2.10	1.19	.235	.13 [-.09, .36]
Group(Young):SWLS	-2.41	2.33	-1.03	.302	-.12 [-.34, .11]
		Neutral as reference			
Positive vs Neutral					
(Intercept)	1.39	0.82	1.70	.089	.19 [-.03, .41]
Group(Young)	-1.25	1.03	-1.21	.226	-.14 [-.36, .08]
PSS	0.29	1.25	0.23	.816	.03 [-.20, .25]
PANAS-NEG	1.42	1.30	1.09	.274	.12 [-.10, .35]
PANAS-POS	0.86	0.76	1.13	.257	.13 [-.09, .35]
SWLS	1.54	0.96	1.60	.109	.18 [-.04, .40]
Group(Young):PSS	-0.87	1.57	-0.55	.580	-.06 [-.29, .16]
Group(Young):PANAS-NEG	-2.33	1.51	-1.54	.124	-.17 [-.39, .05]
Group(Young):PANAS-POS	-0.06	1.00	-0.06	.955	-.01 [-.23, .22]
Group(Young):SWLS	-1.41	1.28	-1.10	.272	-.12 [-.35, .10]

Note: significant ($p < .05$) are bolded for ease of interpretation; SE = standard error of the estimate; p = p-value, r = effect size; PSS: Perceived Stress Scale; PANAS-NEG: Positive and Negative Affect Schedule - Negative Affect; PANAS-POS: Positive and Negative Affect Schedule - Positive Affect; SWLS: Satisfaction With Life Scale.

Table S2. Results of the multilevel multinomial regression for the emotional valence of all statements

Predictor	Estimate	SE	z-statistic	p	r
			Negative as reference		
Neutral vs Negative					
(Intercept)	0.43	0.31	1.39	.164	.16 [-.06, .38]
**Group(Young)**	**0.87**	**0.44**	**1.98**	**.048**	**.22 [.01, .44]**
PSS	-0.40	0.47	-0.86	.392	-.10 [-.32, .13]
PANAS-NEG	0.40	0.51	0.79	.433	.09 [-.13, .31]
**PANAS-POS**	**-1.09**	**0.40**	**-2.73**	**.006**	**-.30 [-.51, -.09]**
**SWLS**	**0.91**	**0.33**	**2.74**	**.006**	**.30 [.09, .51]**
Group(Young):PSS	0.06	0.77	0.07	.942	.01 [-.22, .23]
Group(Young):PANAS-NEG	-0.80	0.64	-1.26	.207	-.14 [-.36, .08]
**Group(Young):PANAS-POS**	**1.75**	**0.55**	**3.20**	**.001**	**.34 [.15, .54]**
Group(Young):SWLS	-0.66	0.52	-1.27	.206	-.14 [-.37, .08]
Positive vs Negative					
(Intercept)	0.62	0.33	1.90	.058	.21 [.00, .43]
Group(Young)	0.84	0.46	1.83	.068	.21 [-.01, .42]
PSS	-0.17	0.51	-0.34	.733	-.04 [-.27, .19]
PANAS-NEG	-0.16	0.54	-0.29	.775	-.03 [-.26, .19]
PANAS-POS	-0.56	0.43	-1.30	.192	-.15 [-.37, .07]
**SWLS**	**0.99**	**0.35**	**2.78**	**.005**	**.30 [.10, .51]**
Group(Young):PSS	-0.42	0.80	-0.53	.596	-.06 [-.29, .16]
Group(Young):PANAS-NEG	-0.49	0.68	-0.73	.467	-.08 [-.31, .14]
**Group(Young):PANAS-POS**	**1.28**	**0.58**	**2.21**	**.027**	**.25 [.03, .46]**
Group(Young):SWLS	-0.52	0.56	-0.94	.348	-.11 [-.33, .12]
			Neutral as reference		
Positive vs Neutral					
(Intercept)	0.20	0.29	0.68	.495	.08 [-.15, .30]
Group(Young)	0.00	0.37	-0.01	.990	.00 [-.23, .22]
PSS	0.25	0.46	0.54	.591	.06 [-.16, .29]
PANAS-NEG	-0.53	0.48	-1.10	.271	-.13 [-.35, .10]
PANAS-POS	0.50	0.33	1.50	.134	.17 [-.05, .39]
SWLS	0.09	0.30	0.29	.771	.03 [-.19, .26]
Group(Young):PSS	-0.47	0.60	-0.78	.436	-.09 [-.31, .14]
Group(Young):PANAS-NEG	0.31	0.58	0.54	.589	.06 [-.16, .29]
Group(Young):PANAS-POS	-0.44	0.42	-1.04	.300	-.12 [-.34, .11]
Group(Young):SWLS	0.12	0.44	0.28	.779	.03 [-.19, .26]

Note: significant ($p < .05$) are bolded for ease of interpretation; SE = standard error of the estimate; p = p-value, r = effect size; PSS: Perceived Stress Scale; PANAS-NEG: Positive and Negative Affect Schedule - Negative Affect; PANAS-POS: Positive and Negative Affect Schedule - Positive Affect; SWLS: Satisfaction With Life Scale.

Table S3. Results of the multilevel multinomial regression for the emotional valence of all statements considering statement as a fixed effect

Predictor	Estimate	SE	z-statistic	p	R
	Negative as reference				
Neutral vs Negative					
(Intercept)	0.06	0.53	0.11	.913	.01 [-.21, .24]
Group(Young)	0.90	0.48	1.90	.058	.21 [.00, .43]
PSS	-0.43	0.52	-0.83	.404	-.10 [-.32, .13]
PANAS-NEG	0.40	0.56	0.71	.479	.08 [-.14, .31]
**PANAS-POS**	**-1.13**	**0.43**	**-2.61**	**.009**	**-.29 [-.49, -.08]**
**SWLS**	**0.94**	**0.36**	**2.60**	**.009**	**.29 [.08, .49]**
st02	-0.22	0.58	-0.37	.710	-.04 [-.27, .18]
st03	0.28	0.61	0.47	.640	.05 [-.17, .28]
st04	1.07	0.60	1.79	.073	.20 [-.02, .42]
st05	1.19	0.62	1.93	.053	.22 [.00, .43]
st06	-0.37	0.57	-0.64	.525	-.07 [-.30, .15]
st07	0.17	0.60	0.28	.779	.03 [-.19, .26]
st08	1.26	0.69	1.82	.069	.20 [-.01, .42]
st09	0.35	0.62	0.55	.579	.06 [-.16, .29]
st10	0.19	0.61	0.30	.761	.03 [-.19, .26]
Group(Young):PSS	0.12	0.82	0.14	.888	.02 [-.21, .24]
Group(Young):PANAS-NEG	-0.82	0.69	-1.19	.235	-.13 [-.36, .09]
**Group(Young):PANAS-POS**	**1.78**	**0.59**	**3.03**	**.002**	**.33 [.13, .53]**
Group(Young):SWLS	-0.69	0.56	-1.22	.223	-.14 [-.36, .08]
Positive vs Negative					
(Intercept)	0.81	0.52	1.53	.125	.17 [-.05, .39]
Group(Young)	0.87	0.51	1.69	.091	.19 [-.03, .41]
PSS	-0.22	0.58	-0.38	.701	-.04 [-.27, .18]
PANAS-NEG	-0.13	0.62	-0.21	.834	-.02 [-.25, .20]
PANAS-POS	-0.59	0.48	-1.23	.219	-.14 [-.36, .08]
**SWLS**	**1.04**	**0.40**	**2.61**	**.009**	**.29 [.08, .49]**
st02	-0.18	0.54	-0.34	.736	-.04 [-.26, .19]
st03	0.34	0.57	0.60	.551	.07 [-.16, .29]
st04	-0.25	0.60	-0.42	.673	-.05 [-.27, .18]
st05	-0.44	0.62	-0.71	.481	-.08 [-.31, .14]
st06	-0.60	0.54	-1.11	.267	-.13 [-.35, .10]
st07	-0.49	0.58	-0.84	.400	-.10 [-.32, .13]
st08	1.01	0.67	1.50	.134	.17 [-.05, .39]
st09	-0.31	0.60	-0.52	.600	-.06 [-.29, .17]
st10	-0.63	0.59	-1.06	.289	-.12 [-.34, .10]
Group(Young):PSS	-0.40	0.88	-0.46	.647	-.05 [-.28, .17]
Group(Young):PANAS-NEG	-0.52	0.76	-0.68	.495	-.08 [-.30, .15]
**Group(Young):PANAS-POS**	**1.29**	**0.64**	**2.03**	**.042**	**.23 [.01, .44]**
Group(Young):SWLS	-0.57	0.61	-0.93	.352	-.11 [-.33, .12]
	Neutral as reference				
Positive vs Neutral					
(Intercept)	0.72	0.43	1.67	.095	.19 [-.03, .41]
Group(Young)	-0.02	0.41	-0.06	.955	-.01 [-.23, .22]
PSS	0.23	0.51	0.46	.649	.05 [-.17, .28]
PANAS-NEG	-0.51	0.53	-0.95	.340	-.11 [-.33, .11]

PANAS-POS	0.52	0.37	1.39	.163	.16 [-.06, .38]
SWLS	0.11	0.34	0.33	.744	.04 [-.19, .26]
st02	0.04	0.44	0.10	.924	.01 [-.22, .24]
st03	0.05	0.43	0.13	.900	.01 [-.21, .24]
**st04**	**-1.25**	**0.41**	**-3.04**	**.002**	**-.33 [-.53, -.13]**
**st05**	**-1.54**	**0.42**	**-3.71**	**<.001**	**-.39 [-.58, -.20]**
st06	-0.22	0.44	-0.50	.620	-.06 [-.28, .17]
st07	-0.61	0.44	-1.37	.169	-.16 [-.38, .07]
st08	-0.23	0.43	-0.53	.594	-.06 [-.29, .16]
st09	-0.61	0.44	-1.37	.171	-.16 [-.38, .07]
st10	-0.76	0.45	-1.70	.090	-.19 [-.41, .03]
Group(Young):PSS	-0.52	0.67	-0.77	.439	-.09 [-.31, .14]
Group(Young):PANAS-NEG	0.32	0.64	0.50	.616	.06 [-.17, .28]
Group(Young):PANAS-POS	-0.46	0.47	-0.99	.324	-.11 [-.34, .11]
Group(Young):SWLS	0.10	0.48	0.20	.840	.02 [-.20, .25]

Note: significant (*p* < .05) are bolded for ease of interpretation; SE = standard error of the estimate; p = p-value, r = effect size; st# = statement n. #; PSS: Perceived Stress Scale; PANAS-NEG: Positive and Negative Affect Schedule - Negative Affect; PANAS-POS: Positive and Negative Affect Schedule - Positive Affect; SWLS: Satisfaction With Life Scale.

Personal relevance

Table S4. Results of the linear model for the personal relevance of the first statement

Predictor	Estimate	SE	t-statistic (df = 65)	p	r
(Intercept)	4.16	0.25	16.98	<.001	.90 [.86, .95]
Group(Young)	-0.38	0.30	-1.25	.215	-.15 [-.39, .09]
PSS	-0.05	0.38	-0.14	.892	-.02 [-.26, .23]
**PANAS-NEG**	**0.74**	**0.36**	**2.07**	**.043**	**.25 [.02, .48]**
PANAS-POS	0.00	0.25	0.01	.990	.00 [-.24, .25]
**SWLS**	**0.57**	**0.22**	**2.57**	**.013**	**.30 [.08, .53]**
Group(Young):PSS	0.20	0.48	0.42	.679	.05 [-.19, .30]
Group(Young):PANAS-NEG	-0.34	0.42	-0.81	.420	-.10 [-.34, .14]
Group(Young):PANAS-POS	0.36	0.34	1.06	.292	.13 [-.11, .37]
Group(Young):SWLS	-0.06	0.32	-0.19	.853	-.02 [-.27, .22]

Note: significant (*p* < .05) are bolded for ease of interpretation; SE = standard error of the estimate; p = p-value, r = effect size; PSS: Perceived Stress Scale; PANAS-NEG: Positive and Negative Affect Schedule - Negative Affect; PANAS-POS: Positive and Negative Affect Schedule - Positive Affect; SWLS: Satisfaction With Life Scale.

Table S5. Results of the linear mixed model for the personal relevance of all statements considering statement as a random effect

Predictor	Estimate	SE	t-statistic (df = 65)	p	r
(Intercept)	3.77	0.12	30.21	<.001	.97 [.96, .99]
Group(Young)	0.09	0.16	0.59	.560	.00 [-.25, .25]
PSS	-0.01	0.19	-0.05	.959	-.01 [-.27, .26]
PANAS-NEG	0.06	0.20	0.30	.762	.04 [-.23, .31]
PANAS-POS	0.16	0.14	1.14	.258	.15 [-.11, .42]
SWLS	0.13	0.12	1.03	.308	.14 [-.12, .40]
Group(Young):PSS	-0.15	0.26	-0.57	.572	-.07 [-.32, .17]
Group(Young):PANAS-NEG	0.08	0.24	0.34	.737	.04 [-.21, .29]
Group(Young):PANAS-POS	-0.07	0.18	-0.41	.687	-.05 [-.29, .19]
Group(Young):SWLS	-0.20	0.18	-1.11	.272	-.13 [-.36, .10]

Note: SE = standard error of the estimate; p = p-value, r = effect size; PSS: Perceived Stress Scale; PANAS-NEG: Positive and Negative Affect Schedule - Negative Affect; PANAS-POS: Positive and Negative Affect Schedule - Positive Affect; SWLS: Satisfaction With Life Scale.

Table S6. Results of the linear mixed model for personal relevance of all statements considering statement as a fixed effect

Predictor	Estimate	SE	t-statistic (df = 65)	p	r
(Intercept)	3.75	0.15	25.44	<.001	.93 [.90, .95]
Group(Young)	0.09	0.16	0.59	.559	.07 [-.18, .32]
PSS	-0.01	0.19	-0.05	.957	-.01 [-.27, .26]
PANAS-NEG	0.06	0.20	0.31	.761	.04 [-.23, .31]
PANAS-POS	0.16	0.14	1.14	.258	.15 [-.11, .42]
SWLS	0.13	0.12	1.03	.308	.14 [-.12, .40]
st02	0.05	0.12	0.40	.692	.02 [-.06, .10]
st03	0.02	0.12	0.21	.837	.01 [-.07, .09]
st04	0.17	0.12	1.45	.149	.06 [-.02, .14]
st05	-0.07	0.12	-0.58	.562	-.02 [-.10, .06]
st06	-0.17	0.12	-1.41	.159	-.06 [-.14, .02]
st07	0.05	0.12	0.39	.698	.02 [-.06, .09]
st08	0.07	0.12	0.56	.578	.02 [-.06, .10]
st09	0.02	0.12	0.19	.852	.01 [-.07, .09]
st10	0.02	0.12	0.20	.842	.01 [-.07, .09]
Group(Young):PSS	-0.15	0.26	-0.57	.571	-.07 [-.32, .17]
Group(Young):PANAS-NEG	0.08	0.24	0.34	.734	.04 [-.21, .29]
Group(Young):PANAS-POS	-0.07	0.18	-0.40	.691	-.05 [-.29, .19]
Group(Young):SWLS	-0.20	0.18	-1.10	.277	-.13 [-.36, .10]

Note: SE = standard error of the estimate; p = p-value, r = effect size; PSS: Perceived Stress Scale; PANAS-NEG: Positive and Negative Affect Schedule - Negative Affect; PANAS-POS: Positive and Negative Affect Schedule - Positive Affect; SWLS: Satisfaction With Life Scale.

Categories of self-images

Table S7. Results of the multilevel multinomial regression for content category of all statements

Predictor	Estimate	SE	z-statistic	p	r
Social identities as reference					
Emotional states vs Social Identities					
(Intercept)	1.40	0.67	2.08	.037	.24 [.02, .45]
Group(Young)	-0.16	0.98	-0.16	.873	-.02 [-.25, .21]
PSS	-0.33	0.90	-0.37	.711	-.04 [-.27, .19]
PANAS-NEG	1.24	1.02	1.22	.223	.14 [-.08, .37]
PANAS-POS	1.00	0.69	1.45	.147	.17 [-.06, .39]
SWLS	-0.44	0.57	-0.77	.439	-.09 [-.32, .14]
Group(Young):PSS	0.71	1.42	0.50	.617	.06 [-.17, .29]
Group(Young):PANAS-NEG	-0.44	1.36	-0.33	.745	-.04 [-.27, .19]
Group(Young):PANAS-POS	-1.11	1.02	-1.08	.279	-.12 [-.35, .10]
Group(Young):SWLS	0.59	1.00	0.59	.559	.07 [-.16, .30]
Specific attributes vs Social Identities					
(Intercept)	-0.14	0.99	-0.14	.888	-.02 [-.25, .21]
Group(Young)	0.52	1.31	0.40	.692	.05 [-.18, .27]
PSS	0.30	1.28	0.23	.816	.03 [-.20, .26]
PANAS-NEG	-0.04	1.48	-0.03	.977	.00 [-.23, .23]
PANAS-POS	0.00	0.98	0.00	.997	.00 [-.23, .23]
SWLS	0.33	0.80	0.41	.681	.05 [-.18, .28]
Group(Young):PSS	0.93	1.82	0.51	.608	.06 [-.17, .29]
Group(Young):PANAS-NEG	0.35	1.83	0.19	.851	.02 [-.21, .25]
Group(Young):PANAS-POS	0.59	1.31	0.45	.654	.05 [-.18, .28]
Group(Young):SWLS	0.21	1.31	0.16	.873	.02 [-.21, .25]
Traits vs Social Identities					
(Intercept)	2.12	0.51	4.13	<.001	.43 [.25, .62]
Group(Young)	1.28	0.79	1.63	.104	.19 [-.04, .41]
PSS	-0.73	0.62	-1.18	.238	-.14 [-.36, .09]
PANAS-NEG	1.10	0.76	1.45	.147	.17 [-.06, .39]
PANAS-POS	0.79	0.49	1.62	.105	.19 [-.04, .41]
SWLS	-0.20	0.40	-0.50	.616	-.06 [-.29, .17]
Group(Young):PSS	0.87	1.05	0.83	.409	.10 [-.13, .32]
Group(Young):PANAS-NEG	-0.65	1.06	-0.61	.543	-.07 [-.30, .16]
Group(Young):PANAS-POS	-1.11	0.79	-1.40	.162	-.16 [-.38, .06]
Group(Young):SWLS	1.13	0.77	1.47	.141	.17 [-.05, .39]
Specific attributes as reference					
Emotional states vs Specific attributes					
(Intercept)	1.08	0.57	1.89	.058	.00 [.00, .00]
Group(Young)	-0.99	0.74	-1.33	.184	-.15 [-.38, .07]
PSS	-0.52	0.77	-0.68	.499	-.08 [-.31, .15]
PANAS-NEG	1.22	0.87	1.41	.160	.16 [-.06, .38]
PANAS-POS	0.96	0.58	1.65	.098	.19 [-.03, .41]
SWLS	-0.64	0.48	-1.34	.181	-.15 [-.38, .07]
Group(Young):PSS	-0.32	1.12	-0.28	.777	-.03 [-.26, .20]
Group(Young):PANAS-NEG	-0.55	1.07	-0.52	.605	-.06 [-.29, .17]
**Group(Young):PANAS-POS**	**-1.66**	**0.79**	**-2.10**	**.036**	**-.24 [-.45, -.02]**
Group(Young):SWLS	0.30	0.79	0.38	.707	.04 [-.19, .27]
Traits vs Specific attributes					

(Intercept)	1.80	0.47	3.83	<.001	.41 [.22, .60]
Group(Young)	0.52	0.59	0.90	.370	.10 [-.12, .33]
PSS	-0.93	0.60	-1.56	.119	-.18 [-.40, .04]
PANAS-NEG	1.07	0.70	1.54	.124	.18 [-.05, .40]
PANAS-POS	0.77	0.45	1.70	.090	.19 [-.03, .41]
SWLS	-0.40	0.37	-1.11	.269	-.13 [-.35, .10]
Group(Young):PSS	-0.19	0.79	-0.24	.809	-.03 [-.26, .20]
Group(Young):PANAS-NEG	-0.76	0.83	-0.91	.362	-.11 [-.33, .12]
**Group(Young):PANAS-POS**	**-1.68**	**0.58**	**-2.92**	**.004**	**-.32 [-.53, -.12]**
Group(Young):SWLS	0.77	0.58	1.33	.184	.15 [-.07, .38]
		Emotional states as reference			
Traits vs Emotional states					
(Intercept)	0.60	0.52	1.15	.249	.13 [-.09, .36]
**Group(Young)**	**1.31**	**0.66**	**1.99**	**.047**	**.23 [.01, .44]**
PSS	-0.42	0.78	-0.54	.587	-.06 [-.29, .17]
PANAS-NEG	-0.10	0.82	-0.12	.906	-.01 [-.24, .22]
PANAS-POS	-0.10	0.59	-0.17	.868	-.02 [-.25, .21]
SWLS	0.20	0.50	0.41	.684	.05 [-.18, .28]
Group(Young):PSS	0.15	1.07	0.14	.887	.02 [-.21, .25]
Group(Young):PANAS-NEG	-0.09	0.98	-0.09	.925	-.01 [-.24, .22]
Group(Young):PANAS-POS	-0.13	0.75	-0.18	.860	-.02 [-.25, .21]
Group(Young):SWLS	0.52	0.74	0.70	.486	.08 [-.15, .31]

Note: significant ($p < .05$) are bolded for ease of interpretation; SE = standard error of the estimate; p = p-value, r = effect size; PSS: Perceived Stress Scale; PANAS-NEG: Positive and Negative Affect Schedule - Negative Affect; PANAS-POS: Positive and Negative Affect Schedule - Positive Affect; SWLS: Satisfaction With Life Scale.

Table S8. Results of the multilevel multinomial regression for content category of all statements considering statement as a fixed effect

Predictor	Estimate	SE	z-statistic	p	r
		Social identities as reference			
Emotional states vs Social Identities					
(Intercept)	0.80	0.83	0.97	.334	.11 [-.12, .34]
Group(Young)	0.03	0.99	0.03	.976	.00 [-.23, .23]
PSS	-0.29	0.92	-0.31	.757	-.04 [-.27, .19]
PANAS-NEG	1.14	1.04	1.10	.272	.13 [-.10, .35]
PANAS-POS	1.04	0.70	1.48	.140	.17 [-.05, .39]
SWLS	-0.56	0.59	-0.96	.339	-.11 [-.34, .12]
st02	0.24	0.75	0.32	.747	.04 [-.19, .27]
st03	-0.72	0.79	-0.90	.367	-.10 [-.33, .12]
st04	0.54	0.78	0.69	.492	.08 [-.15, .31]
st05	0.83	0.86	0.97	.334	.11 [-.11, .34]
st06	0.74	0.96	0.77	.442	.09 [-.14, .32]
**st07**	**2.56**	**1.24**	**2.07**	**.039**	**.23 [.02, .45]**
st08	0.50	1.04	0.48	.630	.06 [-.17, .28]
st09	1.67	1.26	1.33	.184	.15 [-.07, .38]
st10	0.20	1.40	0.14	.887	.02 [-.21, .25]
Group(Young):PSS	0.72	1.46	0.49	.623	.06 [-.17, .29]

Group(Young):PANAS-NEG	-0.40	1.37	-0.29	.770	-.03 [-.26, .20]
Group(Young):PANAS-POS	-1.18	1.06	-1.12	.263	-.13 [-.35, .10]
Group(Young):SWLS	0.69	1.02	0.67	.501	.08 [-.15, .31]
Specific attributes vs Social Identities					
(Intercept)	-3.00	1.35	-2.22	.026	-.25 [-.47, -.03]
Group(Young)	0.96	1.35	0.71	.477	.08 [-.15, .31]
PSS	0.48	1.34	0.36	.719	.04 [-.19, .27]
PANAS-NEG	-0.35	1.54	-0.23	.818	-.03 [-.26, .20]
PANAS-POS	0.03	1.02	0.03	.976	.00 [-.23, .23]
SWLS	0.20	0.83	0.24	.811	.03 [-.20, .26]
st02	1.38	1.11	1.24	.216	.14 [-.08, .37]
st03	1.75	1.07	1.63	.103	.19 [-.04, .41]
**st04**	**2.37**	**1.11**	**2.14**	**.032**	**.24 [.03, .46]**
**st05**	**2.84**	**1.14**	**2.48**	**.013**	**.28 [.07, .49]**
**st06**	**3.51**	**1.20**	**2.94**	**.003**	**.32 [.12, .53]**
**st07**	**4.29**	**1.45**	**2.95**	**.003**	**.32 [.12, .53]**
**st08**	**3.71**	**1.24**	**3.00**	**.003**	**.33 [.12, .53]**
**st09**	**4.50**	**1.44**	**3.13**	**.002**	**.34 [.14, .54]**
**st10**	**3.85**	**1.49**	**2.58**	**.010**	**.29 [.08, .50]**
Group(Young):PSS	0.72	1.88	0.38	.702	.04 [-.18, .27]
Group(Young):PANAS-NEG	0.61	1.89	0.32	.747	.04 [-.19, .27]
Group(Young):PANAS-POS	0.49	1.36	0.36	.721	.04 [-.19, .27]
Group(Young):SWLS	0.28	1.35	0.21	.833	.02 [-.20, .25]
Traits vs Social Identities					
(Intercept)	1.05	0.64	1.63	.104	.19 [-.04, .41]
**Group(Young)**	**1.62**	**0.78**	**2.09**	**.037**	**.24 [.02, .45]**
PSS	-0.65	0.61	-1.06	.289	-.12 [-.35, .10]
PANAS-NEG	0.92	0.73	1.26	.209	.14 [-.08, .37]
PANAS-POS	0.79	0.47	1.66	.098	.19 [-.03, .41]
SWLS	-0.30	0.39	-0.76	.445	-.09 [-.32, .14]
st02	0.40	0.64	0.62	.533	.07 [-.16, .30]
st03	0.33	0.62	0.53	.600	.06 [-.17, .29]
st04	0.43	0.68	0.63	.531	.07 [-.16, .30]
st05	1.05	0.74	1.43	.153	.16 [-.06, .39]
st06	1.52	0.83	1.84	.066	.21 [-.01, .43]
st07	2.07	1.17	1.77	.077	.20 [-.02, .42]
st08	1.67	0.90	1.86	.062	.21 [-.01, .43]
**st09**	**2.38**	**1.16**	**2.06**	**.040**	**.23 [.02, .45]**
**st10**	**2.34**	**1.17**	**2.01**	**.045**	**.23 [.01, .44]**
Group(Young):PSS	0.81	1.05	0.77	.441	.09 [-.14, .32]
Group(Young):PANAS-NEG	-0.53	1.04	-0.51	.611	-.06 [-.29, .17]
Group(Young):PANAS-POS	-1.15	0.80	-1.45	.148	-.17 [-.39, .06]
Group(Young):SWLS	1.24	0.76	1.63	.103	.19 [-.04, .41]
Specific attributes as reference					
Emotional states vs Specific attributes					
(Intercept)	3.12	1.02	3.05	.002	.33 [.13, .54]
Group(Young)	-1.26	0.78	-1.61	.107	-.18 [-.41, .04]
PSS	-0.65	0.81	-0.80	.422	-.09 [-.32, .13]
PANAS-NEG	1.46	0.92	1.58	.113	.18 [-.04, .40]
PANAS-POS	1.00	0.61	1.63	.104	.19 [-.04, .41]
SWLS	-0.66	0.50	-1.31	.190	-.15 [-.37, .07]

st02	-1.01	1.02	-0.99	.323	-.11 [-.34, .11]
**st03**	**-2.22**	**1.04**	**-2.14**	**.033**	**-.24 [-.46, -.02]**
st04	-1.60	0.98	-1.64	.102	-.19 [-.41, .03]
st05	-1.75	0.99	-1.76	.078	-.20 [-.42, .02]
**st06**	**-2.37**	**1.01**	**-2.34**	**.019**	**-.26 [-.48, -.05]**
st07	-1.44	0.99	-1.46	.144	-.17 [-.39, .06]
**st08**	**-2.91**	**1.03**	**-2.83**	**.005**	**-.31 [-.52, -.11]**
**st09**	**-2.54**	**1.01**	**-2.51**	**.012**	**-.28 [-.49, -.07]**
**st10**	**-3.40**	**1.21**	**-2.81**	**.005**	**-.31 [-.52, -.10]**
Group(Young):PSS	-0.13	1.17	-0.11	.915	-.01 [-.24, .22]
Group(Young):PANAS-NEG	-0.77	1.13	-0.69	.492	-.08 [-.31, .15]
**Group(Young):PANAS-POS**	**-1.63**	**0.82**	**-1.97**	**.049**	**-.22 [-.44, -.01]**
Group(Young):SWLS	0.28	0.82	0.35	.730	.04 [-.19, .27]
Traits vs Specific attributes					
(Intercept)	3.28	0.96	3.41	.001	.37 [.17, .57]
Group(Young)	0.44	0.68	0.65	.516	.08 [-.15, .30]
PSS	-1.00	0.71	-1.41	.158	-.16 [-.39, .06]
PANAS-NEG	1.19	0.82	1.45	.148	.17 [-.06, .39]
PANAS-POS	0.81	0.54	1.50	.135	.17 [-.05, .39]
SWLS	-0.43	0.44	-0.98	.326	-.11 [-.34, .11]
st02	-0.82	0.95	-0.87	.387	-.10 [-.33, .13]
st03	-1.16	0.92	-1.27	.205	-.15 [-.37, .08]
st04	-1.67	0.91	-1.84	.067	-.21 [-.43, .01]
st05	-1.47	0.91	-1.62	.106	-.18 [-.41, .04]
st06	-1.55	0.89	-1.73	.084	-.20 [-.42, .02]
**st07**	**-1.84**	**0.91**	**-2.02**	**.044**	**-.23 [-.45, -.01]**
st08	-1.66	0.89	-1.87	.062	-.21 [-.43, .01]
st09	-1.73	0.90	-1.93	.054	-.22 [-.44, .00]
st10	-1.14	0.95	-1.20	.229	-.14 [-.36, .09]
Group(Young):PSS	-0.08	0.94	-0.09	.932	-.01 [-.24, .22]
Group(Young):PANAS-NEG	-0.84	0.97	-0.86	.389	-.10 [-.33, .13]
**Group(Young):PANAS-POS**	**-1.66**	**0.68**	**-2.44**	**.015**	**-.27 [-.49, -.06]**
Group(Young):SWLS	0.79	0.68	1.17	.242	.13 [-.09, .36]
			Emotional states as reference		
Traits vs Emotional states					
(Intercept)	0.08	0.49	0.16	.877	.02 [-.21, .25]
**Group(Young)**	**1.48**	**0.46**	**3.20**	**.001**	**.35 [.15, .55]**
PSS	-0.42	0.52	-0.79	.427	-.09 [-.32, .14]
PANAS-NEG	-0.11	0.55	-0.21	.835	-.02 [-.25, .20]
PANAS-POS	-0.20	0.40	-0.50	.616	-.06 [-.29, .17]
SWLS	0.25	0.34	0.75	.453	.09 [-.14, .31]
st02	0.16	0.52	0.31	.754	.04 [-.19, .27]
st03	0.99	0.60	1.64	.102	.19 [-.03, .41]
st04	-0.07	0.53	-0.12	.901	-.01 [-.24, .21]
st05	0.33	0.56	0.59	.559	.07 [-.16, .30]
st06	0.86	0.60	1.43	.153	.16 [-.06, .39]
st07	-0.26	0.57	-0.46	.649	-.05 [-.28, .18]
st08	1.19	0.65	1.84	.066	.21 [-.01, .43]
st09	0.79	0.62	1.28	.201	.15 [-.08, .37]
**st10**	**2.12**	**0.86**	**2.48**	**.013**	**.28 [.06, .49]**
Group(Young):PSS	0.07	0.78	0.10	.925	.01 [-.22, .24]

Group(Young):PANAS-NEG	-0.10	0.69	-0.15	.883	-.02 [-.25, .21]
Group(Young):PANAS-POS	-0.11	0.56	-0.19	.848	-.02 [-.25, .21]
Group(Young):SWLS	0.56	0.56	1.00	.319	.12 [-.11, .34]

Note: significant ($p < .05$) are bolded for ease of interpretation; SE = standard error of the estimate; p = p-value, r = effect size; st# = statement n. #; PSS: Perceived Stress Scale; PANAS-NEG: Positive and Negative Affect Schedule - Negative Affect; PANAS-POS: Positive and Negative Affect Schedule - Positive Affect; SWLS: Satisfaction With Life Scale.
